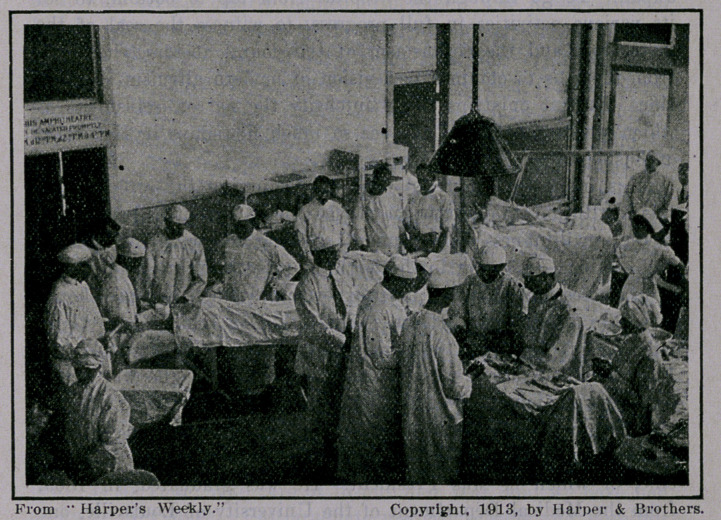# Wonderful Achievements of the New York Polyclinic Medical School and Hospital

**Published:** 1913-08

**Authors:** William Inglis


### Wonderful Achievements of the New York Polyclinic Medical School and Hospital.

BY WILLIAM INGLIS.

If a young doctor could begin practice where an old doctor leaves off—that is, if he could possess in addition to his young strength and zeal and stamina the ripe experience and familiarity with disease and cure that the old doctor has slowly and laboriously accumulated, the young doctor thus reinforced would be the ideal physician or surgeon.

From "Harper's Weekly." Copyright, 1913, by Harper & Brothers.

To supply this knowledge, to equip the young practitioner with the wisdom acquired by ages of struggle with evil conditions, is the chief ambition of the medical profession today; and perhaps nowhere else in this country are the means of imparting this wisdom together with practical experience so efficiently developed as in the New York Polyclinic Medical School and Hospital. In its spacious new building, twelve stories high, at Nos. 341 to 351 West Fiftieth Street, New York, the work of healing is being carried on in the most efficient manner known to medical and surgical science, while daily in the dispensary, the hospital ward, the operating room, or the laboratory hundreds of young doctors are acquiring a wealth of instruction from the ablest practitioners and of actual contact with cases that sends them back to their neighborhood not only splendidly equipped, but the bearers of the torch of knowledge to others. The newest improvements, discoveries, and inventions in medical and surgical science are all taught here. It is impossible to exaggerate the beneficent influence exerted by such an institution as this not only upon the 100,000 patients there treated every year, but upon the welfare of the country at large. The methods used for their cure are disseminated throughout the length and breadth of the land. In the last thirty years more than 20,000 students have attended the clinics in this institution from the United States, Canada, Mexico, the South American States, the West Indies, Australia, China, and Japan. To go through the hospital from top to bottom, to see its various activities in full progress, to witness the zeal of the instructors and the enthusiasm of the young doctors who learn from them, is to obtain a new vision of modern altruism and sacrifice that far outstrips in its intensity the narrow selfishness of which we hear so much and see so much nowadays in the world of business.

It is interesting to trace from its beginning the evolution of this institution, to discover, in the first place, that it had its origin in the unwillingness of its founder, a young doctor duly graduated and certified, to go out in the world and use suffering mankind as so much research material until experience at their expense should give him proficiency. He was—and is—Dr. John A. Wyeth, a lad who left school in 1861 and fought through the Civil War in Forrest's Confederate Cavalry, and at the end of the war began to study medicine. I quote from the history as Dr. Wyeth related it in an address to the Association of American Medical Colleges on March 20, 1909, at the New York Academy of Medicine, of which he was President. He was graduated, in 1869, from the Medical Department of the University of Louisville, one of the oldest and deservedly best known of the medical colleges in the United States, and yet "the teaching of surgery and medicine was almost wholly didactic. When an operative clinic was given, the students witnessed it at such a distance from the subject and with so many interruptions of vision that it was impossible to follow closely the details of technique, without which the lesson of a demonstration is valueless. Not once in my two college years did I enter the ward of a hospital or receive instruction by the bedside of a patient. * * *

"In my native village of Northern Alabama I put out my sign, but two months of hopeless struggle with a Presbyterian conscience convinced me that I was not fit to practice medicine, and that nothing was left for me but to go out into the world of business to earn money enough to finish my education. I felt the absolute need of clinical experience, and a conviction which then forced itself upon me—that no graduate in medicine is competent to practice until he has in addition to his theoretical a clinical and laboratory training—was the controlling idea in my mind when in later years the opportunity offered and it fell to my good fortune to establish in this city the New York Polyclinic Medical School and Hospital."

The institution was organized in 1881, and in 1882 opened its doors in East Thirty-fourth Street, where for years it ministered to the suffering of the poor and held its place as the pioneer post-graduate medical institution in the United States. At the basis of it were certain clearly defined principles: The preservation of human life against disease, the betterment and upbuilding of mankind, is the first duty of all. Ours is a new country. It is unique. No older civilization is like it. It is a frontier country, with millions of its people scattered in inaccessible regions, with physicians ill prepared to protect them as they should be protected. The founder of the school was graduated under the best conditions of his day, yet felt that his equipment was inefficient. It occurred to him that something must be done to elevate the standard of the medical profession, to stimulate graduates to endeavor to become scientific, practical, proficient doctors. There was nothing to do but organize an institution to afford actual practice for doctors already graduated—of whom, even to this day, only one-tenth obtain first practice as internes in hospitals.

"The result," says Dr. Wyeth, "has been a complete revolution. Looking back at the changes in the last thirty years, I can hardly believe it is the same profession. We had as many doctor-students as we could take care of in our first year, and we have had as many ever since. Post-graduate medical schools and hospitals have sprung up in all of the chief cities of this country. In Chicago alone there are five. If one doctor in a town or village took a course in a post-graduate hospital he was sure to talk of it, and as a result the others had to go.

"It took nearly twenty years for the medical organizations to recognize the change by official action. When I was made—to my great surprise—President of the American Medical Association in 1901, that Association began to use its influence to better American medical schools. It organized a Committee on Medical Colleges and began to raise the standard of admission to practically a college degree; compelled every medical college to require a four-year course as a preliminary to graduation, and, in order so far as possible to eradicate commercialism from the medical profession, provided that all the medical schools of the country should become part of a central university system. For example, the four separate medical colleges which formerly existed in Louisville, Kentucky, are now consolidated in one school, which constitutes the Medical Department of the University of Louisville."

Even a layman could not fail to be impressed by the difference between the methods of instruction in the New York Polyclinic and those of the ordinary medical school. True, there were amphitheaters for lectures and certain clinics; but by far the greater part of the clinics were at the actual bedside of the patient, with sections of four or five doctors in close attendance upon the demonstrator, learning from him, in some cases helping him, but, best of all, acquiring from the experience of actually seeing and treating the cases that knowledge which no amount of distant lecturing in the amphitheater could give. The doctor-students at the Polyclinic saw at close range in the operating room all the intricate details of surgical operations and dressings. Some of them, who had qualified after some months as internes in the institution, performed certain operations. In the laboratory with its courses in histology, pathology, bacteriology, clinical microscopy, and clinical chemistry every student enjoyed the advantage of working beside the head of that department. No one is accepted in this branch unless he shows special aptitude for the work and undertakes to go through the full course of study.

Thus far we have been considering conditions that are ideal. Let us now turn to the practical wants, or, rather, to the one great practical want of the institution—money. If the New York Polyclinic Medical School and Hospital had not done a splendid humanitarian work during thirty-one busy years and were not still doing it, its needs would not perhaps command a respectful hearing. But it does seem a reflection upon the intelligence no less than the philanthropy of America that this asylum for the distressed and fountain of the art of healing should remain as it is, cramped and unable to do its best work, by reason of lack of money. The hospital needs money to build an automobile ambulance station and six accident wards. They ought to be built, equipped, and in use at this moment. The ambulances it already owns cover a district inhabited by 360,000 people, and the hospital has treated as many as forty-five accident cases in one day. One-third of the cases now brought in are treated temporarily and of necessity transferred to Bellevue or the city hospitals because there is not room at the Polyclinic to take care of them.

Those so badly injured that they can not be shifted are kept at the Polyclinic. It is important that all the cases brought in should be kept in the institution not only for the best results to the injured, but for the sake of the experience afforded to the doctor-students, who in turn carry the light into their home neighborhoods.

The running expenses of the Polyclinic are about $250,000 a year. These are met by the fees paid by doctor-students and by the wealthier patients among the one hundred thousand who are treated each year. Thanks to the low cost of the ground on which the hospital is built and a low interest rate, the carrying charges are light as possible and no help is needed there. The only things lacking now are the six accident wards and the automobile ambulance garage. To provide these will cost one million dollars. That million ought to be forthcoming as soon as the moneyed men of America learn what a marvelously beneficent work can be accomplished with it.—*Harper's Weekly.*

## Figures and Tables

**Figure f1:**